# Impaired functional capacity of fetal endothelial cells in preeclampsia

**DOI:** 10.1371/journal.pone.0178340

**Published:** 2017-05-23

**Authors:** Lars Brodowski, Jennifer Burlakov, Sarah Hass, Constantin von Kaisenberg, Frauke von Versen-Höynck

**Affiliations:** Department of Obstetrics and Gynecology, Hannover Medical School, Hannover, Germany; Academic Medical Centre, University of Amsterdam, NETHERLANDS

## Abstract

**Objectives:**

Preeclampsia is one of the main contributers to maternal and fetal morbidity and mortality during pregnancy. A history of preeclampsia puts mother and offspring at an increased cardiovascular risk in later life. We hypothesized that at the time of birth functional impairments of fetal endothelial cells can be detected in pregnancies complicated by preeclampsia and that a therapeutic intervention using 1,25 (OH)_2_ vitamin D_3_ can reverse the adverse effects of preeclampsia on cell function.

**Methods:**

Human umbilical vein endothelial cells (HUVEC) were isolated from umbilical cords obtained from preeclamptic (N = 12) and uncomplicated pregnancies (N = 13, control). Placental villous tissue fragments from uncomplicated term pregnancies were incubated in explant culture for 48 h at 2% (hypoxia), 8% or 21% O_2_. Explant conditioned media (CM) was collected and pooled according to oxygen level. We compared the ability of preeclampsia vs. control HUVEC to migrate, proliferate, and form tubule-like networks in a Matrigel assay, in the presence/absence of CM and 1,25(OH)_2_ vitamin D_3_.

**Results:**

HUVEC from preeclamptic pregnancies showed reduced migration (P = 0.04) and tubule formation (P = 0.04), but no change in proliferation (P = 0.16) compared to healthy pregnancies. Placental villous explant CM derived from 2% O_2_ incubations significantly reduced HUVEC migration, when compared to non-CM (P = 0.04). Vitamin D_3_ improved HUVEC function in neither of the groups. There was no significant difference in VEGF gene expression between healthy and preeclamptic pregnancies and no effect of Vitamin D_3_ on VEGF expression.

**Conclusions:**

Reduced functional abilities of fetal endothelial cells from preeclamptic pregnancies suggests that disease pathways, possibly originating from the dysfunctional placenta, negatively impact fetal endothelium. The neutral effect of 1,25(OH)_2_ vitamin D_3_ contrasts with previous findings that vitamin D rescues the poor migration, proliferation and tubule formation exhibited by cord blood fetal endothelial progenitor cells from preeclamptic pregnancies. Further investigations to distinguish pathways by which offspring exposed to preeclampsia are at risk for cardiovascular disease are needed.

## Introduction

Long-term follow-up studies find that offspring from mothers with preeclampsia exhibit alterations of the cardiovascular system that predispose them to a higher risk for adverse cardiovascular events. Exposure to a preeclamptic *in utero* environment increases the systolic and diastolic blood pressure and body mass index (BMI) already in children and young adults [1, 2]. In their teen years, male and female offspring of mothers with preeclampsia display marked vascular dysfunction as evidenced by a higher pulmonary artery pressure, reduced flow-mediated dilation, and increased carotid intima media thickness, the latter a subclinical marker of atherosclerosis [2, 3]. Preeclampsia is associated with an increased risk of stroke [4, 5] and cardiovascular dysfunction [6, 7] in the adult offspring.

It is hypothesized that the associations between preeclampsia and later cardiovascular function relate to the impaired placental development with placental insufficiency and restricted oxygen supply in preeclampsia. The release of pathogenic factors, e.g. antiangiogenic factors and reactive oxygen species, is thought to trigger a systemic oxidative and inflammatory reaction during the pregnancy [8]. However, the impact of such factors on fetal programming remains unclear.

We have shown previously that fetal endothelial colony-forming cells (ECFC), a subgroup of endothelial progenitor cells (EPC) are reduced in cord blood of preeclamptic pregnancies and exhibit reduced migratory, proliferative and angiogenic capabilities *in vitro* [9, 10]. These differences might represent a first sign of impairment of the neonatal cardiovascular system at the time of delivery. In addition to maternal pregnancy outcome history these findings might help to identify infants at risk for cardiovascular dysfunction already at the time of birth.

Vitamin D is known for its important role in calcium homeostasis and bone metabolism, but it also influences the cardiovascular system through unclear mechanisms [11]. A deficiency of vitamin D is associated with increased cardiovascular disease and all-cause mortality, various cardiovascular risk factors and coronary heart disease [11]. Preeclampsia is characterized by changes in vitamin D and calcium metabolism compared to normal pregnancies [12]. Several observational studies have demonstrated a significant relationship between maternal vitamin D deficiency and the incidence of preeclampsia [13, 14]. Moreover, vitamin D supplementation studies showed protective effects on preeclampsia incidence [14, 15]. Not least we recently showed a significant promotion of *in vitro* angiogenesis by 1,25 (OH)_2_ vitamin D_3_ in fetal ECFC derived from preeclamptic pregnancies, suggesting a regulatory role of vitamin D for ECFC function [9].

As the isolation and characterization of ECFC is time and resource intense, and because adverse effects of a preeclamptic intrauterine environment may not be limited to progenitor endothelial populations, we tested whether preeclampsia impairs the angiogenic properties also of human umbilical vein endothelial cells (HUVEC) and if functional characteristics can be improved by vitamin D. HUVEC are predominantly mature endothelial cells, that are used frequently to study the regulation of endothelial cell function, blood vessel repair, the development of atherosclerotic plaques and angiogenesis [16–18]. As with cord blood endothelial progenitor cells, these fetal endothelial cells may be impacted by pathogenic factors released by the placenta.

## Materials and methods

### Patients

The Ethical Committee at Hannover Medical School approved the study (1507 / 2012). Informed written consent was obtained from each patient. HUVEC derived from umbilical veins of 13 healthy women with uncomplicated, normotensive pregnancies (controls) and 12 women with preeclampsia, at 36.1 +/- 3.9 (control) and 34.0 +/- 4.3 (PE) weeks of gestation, delivered by vaginal or Cesarean section, were used. Four out of 12 preeclamptic women developed early-onset (29.4 +/-2.9 weeks of gestation) and eight late-onset (36.3 +/-2.1 weeks of gestation) preeclampsia. All women had singleton pregnancies. Preeclampsia was defined as gestational hypertension and proteinuria beginning after 20 weeks of pregnancy with resolution of clinical symptoms postpartum [19]. Gestational hypertension was defined as persistent, new onset hypertension (absolute blood pressure ≥140 mmHg systolic and/or ≥90 mmHg diastolic) appearing after 20 weeks of gestation. Proteinuria was defined as ≥300 mg per 24-h urine collection, ≥2+ protein on voided urine sample, ≥1+ protein on catheterized urine specimen, or a protein-creatinine ratio of ≥0.3. Women were classified as having an uncomplicated pregnancy if they were without proteinuria and normotensive throughout gestation and if they delivered healthy babies. All study subjects were non-smokers by self-report, and were without clinical history of preexisting renal, vascular, or metabolic disease. Pre-pregnancy weight, self-reported at enrollment, and measured height were used to calculate pre- pregnancy BMI. Maternal race was by self- report at enrollment. Gestational age-specific birth weight percentiles, adjusted for infant sex and race, were based upon data from Hannover Medical Center (Hannover, Germany).

#### Vitamin D_3_ measurement in patient serum and umbilical cord blood

25(OH) Vitamin D_3_ concentrations in the individual maternal serum prior delivery and cord blood serum samples after delivery were determined in the clinical laboratory at Hannover Medical School using a LIAISON 25(OH) Vitamin D_3_ TOTAL Assay (DiaSorin Inc., USA), as per the manufacturers recommendations.

### HUVEC isolation, culture and phenotyping

HUVEC were isolated from umbilical cord according to the protocol of Jaffe et al. with some modificatios [20]. Briefly, umbilical cords were collected immediately after delivery and stored at 4°C until the cells were isolated between one and four days after delivery under sterile conditions. After incubation of the umbilical cord with collagenase enriched cord buffer for 25 min at 37°C the detached endothelial cells were washed in 10 ml growth medium containing 2.44% (v/v) supplements (human recombinant epidermal growth factor, fibroblast growth factor, VEGF, ascorbic acid, hydrocortisone, recombinant insulin-like growth factor in supplier-recommended concentrations, Lonza, Walkersville, MD, USA), 8% fetal calf serum (FCS, Biochrom, Berlin, Germany) and 1.2% penicillin/ streptomycin (Biochrom, Berlin, Germany) and transferred to a T75 cell culture flask. The next day the medium was refreshed. The HUVEC were cultured at 37°C and 5% CO_2_ in T75 cell culture flasks until they were grown to confluence. After trypsinization they were used for phenotyping or frozen in freezing medium containing 92% FCS and 8% DMSO (Sigma Aldrich, Steinheim, Germany) and stored in liquid nitrogen until they were used for experiments. All experiments were performed with HUVEC in passage 2 to 6. For each functional experiment, HUVEC from a preeclamptic and a corresponding control patient were run in tandem. Using flow cytometry the typical endothelial cell phenotype (CD31+, CD90-) was confirmed. During the cell culture assays HUVEC were incubated with 0 nM (vehicle) or 10 nM of 1,25 (OH)_2_ vitamin D_3_ (Sigma Aldrich, St. Louis, MO). The concentration of 1,25 (OH)_2_ vitamin D_3_ was intended to approximate physiological levels in pregnancy [14] and was previously found to rescue ECFC functions in vitro [9, 10, 21].

### Culture of placental villous explants and preparation of conditioned medium

Placental villous explant preparation and culture was carried out according to published protocols with some modifications [22]. Briefly, placentas from eigth uncomplicated, healthy pregnancies delivered by vaginal or Cesarean section were obtained within 10 min of delivery. After removal of the decidua, biopsies were excised from the maternal side of the placenta. The tissue was immediately transported to the laboratory in ice-cold phosphate buffered saline (PBS) containing 2% penicillin/streptomycin. After rinsing the placental pieces in PBS to wash out blood, large vessels and decidua were removed by blunt dissection. Placental villous explants (1–2 mg each in size) were dissected and used for experiments under different oxygen conditions. An average of 50 mg of finely dissected villous tissue was placed into each well of a 12-well plate containing 1.5 ml of Medium 199 (Sigma-Aldrich, St- Louis, MO, USA) supplemented with 2% FCS and 1% penicillin/streptomycin. The plates were incubated under controlled oxygen conditions (2% O_2_, 8% O_2_ and 21% O_2_) in three separate incubator chambers (Xvivo, Biospherix Inc., USA) at 37°C, 5% CO_2_ for 48 h. The conditioned medium (CM) was centrifuged (3,200 rpm, 4°C, 5 min). The cell-free supernatants were pooled according to the oxygen conditions and stored in aliquots at -80°C. As control, M199 medium supplemented with 2% FBS, 1% penicillin/streptomycin (non-conditioned medium, NCM) was employed in the same ratio as CM.

### In vitro angiogenesis assay

We used an *in vitro* angiogenesis assay (endothelial tubule formation in Matrigel) as a test of HUVEC function. In a 96-well plate 8,000 cells per well were seeded in 150 ul treatment medium with 30 ul growth factor reduced Matrigel (BD Biosciences, Bedford, MA). HUVEC from preeclampsia and a corresponding control patient were run in tandem. The experiments were performed in the presence and absence of 10 nM 1,25(OH)_2_ vitamin D_3_. After six hours of incubation at 37°C each well was photographed with a LEICA DMI 6000 B microscope. The formed tubules were quantified using Image-J software (National Institutes of Health). All experiments were performed in triplicate wells from which values were averaged (N = number of experiments).

### Cell migration assay

To analyze HUVEC migratory ability, 10,000 to 50,000 HUVEC were seeded in each well of a 12-well-plate with growth medium containing 2.44% supplements, 8% FCS and 1.2% penicillin/ streptomycin. After reaching confluence the HUVEC monolayer was scratched using a sterile P200 pipette tip to produce a lane free of cells as described before [23]. HUVEC from preeclampsia and a corresponding control patient were run in tandem. The experiments were performed in the presence and absence of 10 nM 1,25(OH)_2_ vitamin D_3_. Additionally, CM from three different oxygen levels or NCM were added at 50% v/v concentration. The experiments were performed in the presence or absence of 10 nM 1,25(OH)_2_ vitamin D_3_. Light microscopic images were obtained immediately after the scratch (T0) and at the end of the experiment after 18h (T18). Migration into the scratch wound was analysed using Image J software and calculated as percentage of wound closure (percentage of original area at T0 that became occupied by cells by migration into the wound area at T18). All experiments were done in quadruplicate wells from which values were averaged.

### Proliferation assay

To determine the proliferative capacity of HUVEC derived from uncomplicated and preeclamptic pregnancies in the presence or absence of 1,25(OH)_2_ vitamin D_3_, 10,000 cells were seeded per well of 24-well culture plates in EGM supplemented with 8% (v/v) FCS and 1% penicillin/streptomycin. Medium was changed the next day and cells were incubated with 0 nM (vehicle) or 10 nM of 1,25 (OH)_2_ vitamin D_3_. After 24 h, 48 h and 72 h of treatment, the cell number was counted in a Neubauer chamber with 1:2 trypan-blue dilution. Population doubling time was calculated as following: log2/ (logNt–logNo), t = time period (h), Nt = number of cells at time t, No = initial cell number.

### RT-PCR for quantification of VEGF gene expression

The RNA isolation of HUVEC was performed according to the protocol of Chomczynski et al. [24] which was slightly modified. The concentration of RNA of each sample was determined spectrophotometrically (BIO photometer, Eppendorf, Germany) at 260/280 nm and aliquots (2 mg) of this RNA were fractionated on agarose/ethidium bromide gels to check RNA integrity.

For the cDNA synthesis 2 ug of RNA was diluted with diethylpyrocarbonate (DEPC) water to a volume of 8 ul and denatured at 68°C for 10 minutes in a thermocycler (PTC 200, Biozym Scientific GmbH, Germany). Then 12 ul of High Capacity cDNA Reverse transcription (RT) master mix were added. This is made up of RT buffer, RT Random primer, deoxyribonucleoside triphosphate (dNTP) mix (100 mM), MultiScribeTMReverse transcriptase (Applied Biosystems, USA) and DEPC. The generated cDNA was initially diluted 1: 2 with DEPC water. In each case, 1.5 ul diluted cDNA and 10.5 ul master mix were pipetted into the appropriate strip tubes (0.1 ml). For each treatment, medium triplets were created and three RT-PCR runs were done for each patient. For normalization, 18S rRNA served as housekeeping gene. Primer sequences used were as follows: VEGF-A forward primer 5-CTGGAGTGTGTGCCCACTGA-3, VEGF-A reverse primer 5-TCCTATGTGCTGGCCTTGGT-3, 18S rRNA forward primer 5-GAGCGAAAGCATTTGCCAAG-3 239, 18S rRNA reverse primer 5—GGCATCGTTTATGGTCGGAA-3). Ct values were automatically generated and relative quantification of gene expression was calculated by standard ΔCt method using the expression of 18S rRNA as reference. Relative expression levels of HUVEC from uncomplicated pregnancies and pregnancies complicated by preeclampsia with and without vitamin D treatment were finally compared.

### Statistical analysis

Demographic data are expressed as means and standard deviation. Experimental data are presented as means and standard error. Distribution was examined using Kolmogorov- Smirnov test. Continuous data were compared with ANOVA, Kruskal-Wallis, unpaired t-test, Mann-Whitney or Wilcoxon-signed rank test, as appropriate. Data were analyzed with Prism 4 software package (GraphPad Software Inc., La Jolla, CA). Where specified, to account for interassay variation, data are given as fold change in functional variables relative to untreated HUVEC from uncomplicated pregnancies or relative to untreated HUVEC from preeclamptic pregnancies.

## Results

### Patient demographics

The clinical and demographic data for the pregnant women who provided umbilical cord for the analysis of HUVEC function are given in Table 1. Maternal age, maternal pre-pregnancy BMI, race and parity, and baby sex were not statistically different between the preeclampsia and control groups. The percentage of women who delivered by Cesarean section versus vaginal delivery did not differ by outcome group. By definition, women with preeclampsia had higher systolic and diastolic blood pressures at delivery compared to the uncomplicated study group. Patients were matched by gestational age for the cell culture experiments.

**Table 1:** Clinical and demographic data of patients who provided maternal blood samples (data are mean +/- SD).

	Uncomplicated pregnancy (n = 13)	Preeclampsia (n = 12)	P value
Maternal age	32.2 +/-5.2	31.1 +/-4.1	0.55
Gestational age at delivery (wks)	36.1 +/-3.9	34.0 +/-4.3	0.23
Multiparous- n (%)	5 (38%)	3 (25%)	0.70
Maternal pre-pregnancy BMI (kg/m2)	24.9 +/-5.3	26.6 +/-6.3	0.51
Gestational SBP, pre-delivery (mm Hg)	117 +/-13.0	170 +/-19.6	<0.0001
Gestational SBP before 20 week gestation (mm Hg)	119 +/-9.2	127 +/-14.1	0.054
Gestational DBP, pre-delivery (mm Hg)	67.1 +/-7.9	99.6 +/-15.6	<0.0001
Gestational DBP before 20 week gestation (mm Hg)	72.9 +/-8.9	80.6 +/-14.0	0.12
Birth weight (g)	2637 +/- 1026	1907 +/- 857	0.08
Birth weight percentile	34.8 +/- 26.2	20.9 +/- 25.6	0.08
Birth weight percentile <10th- n (%)	3 (23%)	6 (50%)	0.48
Caesarean delivery- n (%)	9 (69%)	9 (75%)	1.0
Maternal Race, Black- n (%)	0 (0%)	0 (0%)	1.0
Baby sex, male- n (%)	9 (69%)	4 (33%)	0.49

### Vitamin D_3_ concentrations in maternal serum and umbilical cord blood

**Fig 1:**
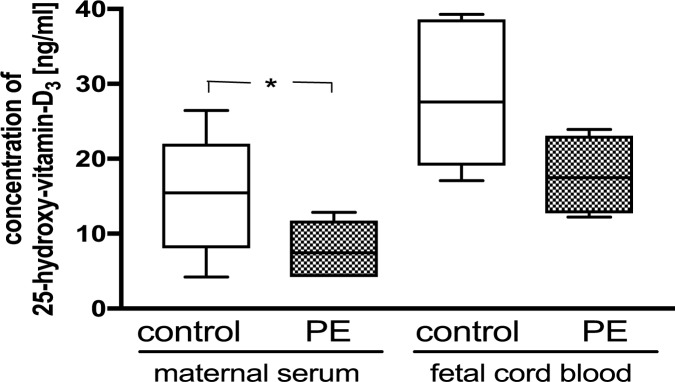
25(OH) vitamin D concentrations (ng/ml) in the individual maternal serum samples prior delivery and umbilical cord blood after delivery. *P < 0.05 vs. control. The ends of the whiskers represent the maximum and minimum measured values. Distribution was examined using Kolmogorov- Smirnov test. Continuous data were compared with unpaired t-test.

Women with preeclampsia had significantly lower 25(OH) vitamin D_3_ levels compared to women with healthy pregnancies (Control: 15.5 +/- 2.4 ng/ml; PE: 7.5 +/- 1.5 ng/ml; P = 0.04), (Fig 1). The 25(OH) vitamin D_3_ levels in umbilical cord blood of preeclamptic pregnancies were marginally lower compared to cord blood of control patients (Control: 27.6 +/- 2.3 ng/ml; PE: 17.5 +/- 2.7 ng/ml; P = 0.06). Umbilical cord blood concentrations of 25(OH) vitamin D_3_ were significantly higher than in maternal sera (Control: maternal vs. umbilical cord blood: P = 0.005; preeclampsia: maternal vs. umbilical cord blood: P = 0.009). Of all patients, 70% of the control group and 100% of the preeclamptic group were vitamin D deficient when defined as 25(OH) vitamin D_3_ concentrations ≤ 20 ng/ml.

### In vitro angiogenesis

**Fig 2:**
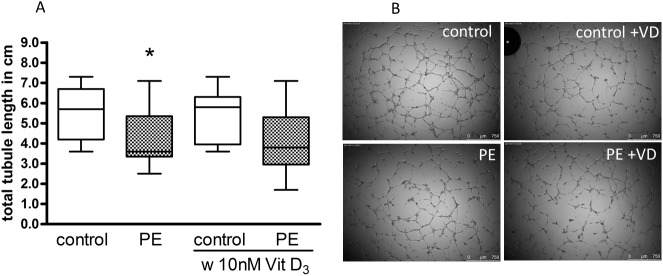
Building of tubule-like structures by uncomplicated pregnancy (control) and preeclampsia (PE) HUVEC, and the effect of 1,25(OH)_2_ vitamin D_3_, on capillary-tube formation by HUVEC in a Matrigel assay. (A) Capillary-tube formation (average total tubule length per microscopic field) was analyzed after 6 h by visual microscopy at 2.5 magnification. Data are expressed as total tubule length in cm. *P < 0.05 vs. control. The ends of the whiskers represent the maximum and minimum measured values. Distribution was examined using Kolmogorov- Smirnov test. Continuous data were compared with Mann-Whitney test. (B) Representative photomicrographs of HUVEC after incubation in Matrigel. Scale bar represents 750 μm.

A Matrigel angiogenesis model was used to assess the capacity of HUVEC to differentiate into tubule-like structures (Fig 2). Tubule assemblage by preeclampsia-derived HUVEC was markedly impaired (Control: 5.5 +/- 0.4 cm; PE: 4.4 +/- 0.4 cm; 72% of control, P = 0.04). The supplementation with 10 nM 1,25(OH)_2_ vitamin D_3_ did not impact the tubule formation of HUVEC from uncomplicated pregnancies (Control: 5.2 +/- 0.4 cm; 96% of control, P = 0.54) or preeclamptic pregnancies (PE: 4.1 +/- 0.5 cm, 94% of control, P = 0.89).

### Migration

**Fig 3:**
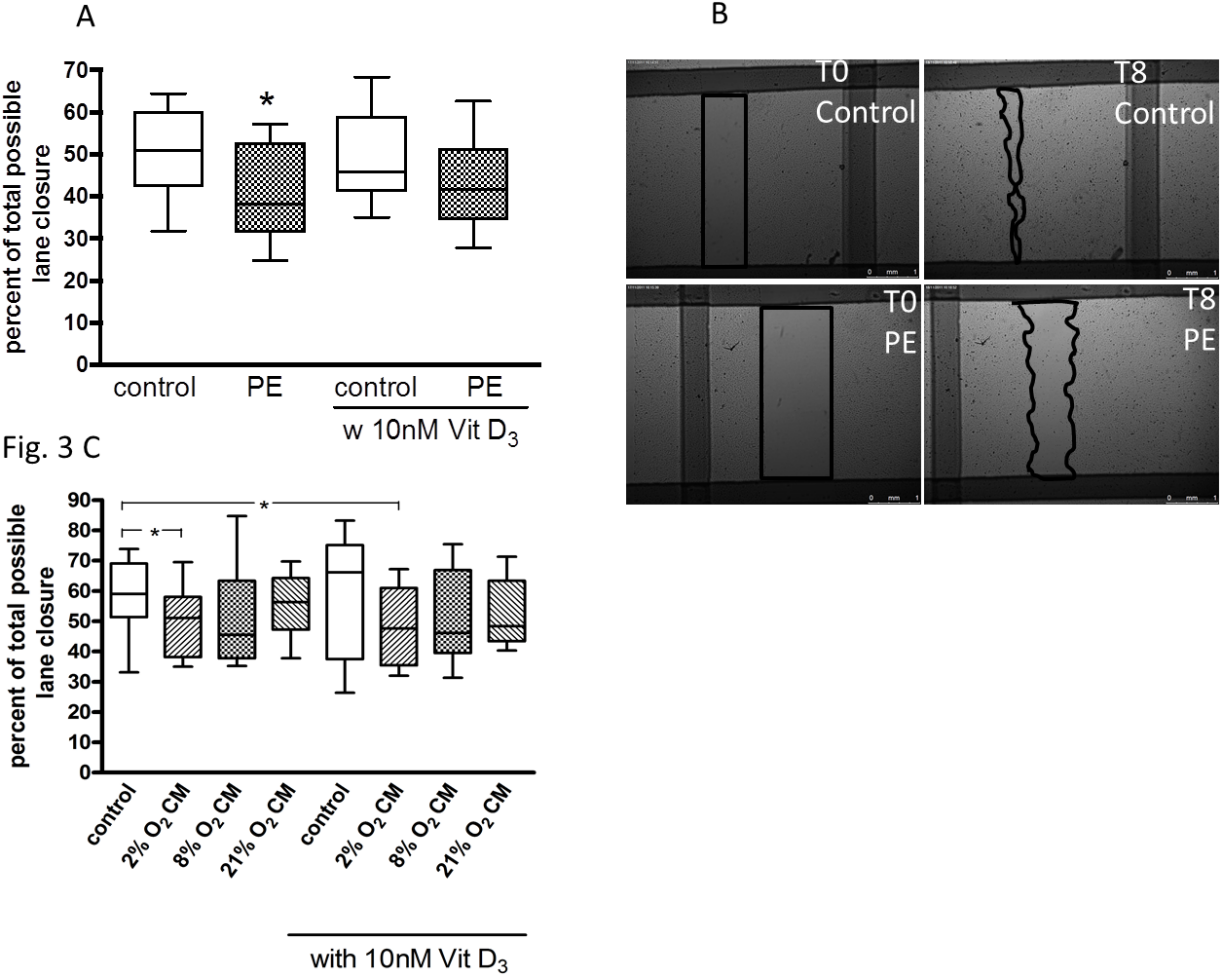
Effect of preeclampsia (PE), preeclampsia-like conditions and 1,25(OH)_2_ vitamin D_3_ on HUVEC migration. (A) HUVEC of uncomplicated (control) and PE pregnancies were cultured in endothelial basal medium (EBM) and treated with or without 10 nM 1,25(OH)_2_ vitamin D_3_. The migration of PE HUVEC into the scratch wound assessed after incubation for 18 h was decreased compared to control. (B) "scratch wound" at 0 h and 8 h incubation is shown. Scale bar represents 750 μm. (C) Effect of villous explant conditioned medium (2% O_2_, 8% O_2_, 21% O_2_ CM) and 1,25(OH)_2_ vitamin D_3_ on HUVEC migration with a reduction at 2% O_2_. *P < 0.05 vs. untreated control. The ends of the whiskers represent the maximum and minimum measured values. Distribution was examined using Kolmogorov- Smirnov test. Continuous data were compared with unpaired t-test (A) or Wilcoxon-signed rank test (C).

With scratch wound area filling expressed as percent of total possible lane closure (100%), the migration of HUVEC from preeclamptic pregnancies (41% +/- 3.2%) was significantly impaired, compared to control (50% +/- 2.9%; P = 0.04) (Fig 3A and 3B). After treatment of HUVEC from healthy and preeclamptic pregnancies with 1,25(OH)_2_ vitamin D_3_ the two groups were no longer significantly different (P = 0.22). However, 1,25(OH)_2_ vitamin D_3_ did not significantly improve HUVEC migration in the control group (49% +/- 2.9%, 92% of control, P = 0.81) or the preeclamptic pregnancy outcome group (44% +/-3.1%, 98% of control, P = 0.53), suggesting at best a marginal impact of vitamin D.

Clinical and demographic data for the uncomplicated pregnant women who provided placental samples for villous explant culture are given in Table 2. Treatment with placental villous explant CM derived from 2% O_2_ incubations led to a reduced HUVEC migration (49% +/- 3.5%, 85% of NCM; P = 0.04), when compared to NCM (59% +/- 3.7%). Compared to NCM, explant CM from 8% O_2_ impaired HUVEC migration, but this effect was not statistically significant (50% +/- 5.2%, 87% of NCM; P = 0.38), (Fig 3C). Explant CM from 21% O_2_ did not affect HUVEC migration (55% +/- 3.1%, 99% of NCM; P = 0.46). Compared to absence of vitamin D, 1,25(OH)_2_ vitamin D_3_ did not improve endothelial cell migration in any CM group.

**Table 2:** Clinical and demographic data for the uncomplicated pregnant women who provided placental samples for villous explant culture (data are mean +/2 SD).

	(n = 8)
Maternal age	33.3 +/-4.9
Gestational age at delivery (wks)	38.2 +/-2.4
Multiparous- n (%)	4 (50%)
Maternal pre-pregnancy BMI (kg/m2)	29.2 +/-9.3
Gestational SBP, pre-delivery (mm Hg)	119.8 +/-11.5
Gestational SBP before 20 week gestation (mm Hg)	75.0 +/-7.6
Birth weight (g)	3626 +/-831
Birth weight percentile	72.3 +/-30.8
Birth weight percentile <10th- n (%)	1 (12.5%)
Caesarean delivery- n (%)	6 (75%)
Maternal Race, Black–n (%)	0 (0%)
Baby gender, male- n (%)	4 (50%)

### Proliferation

**Fig 4:**
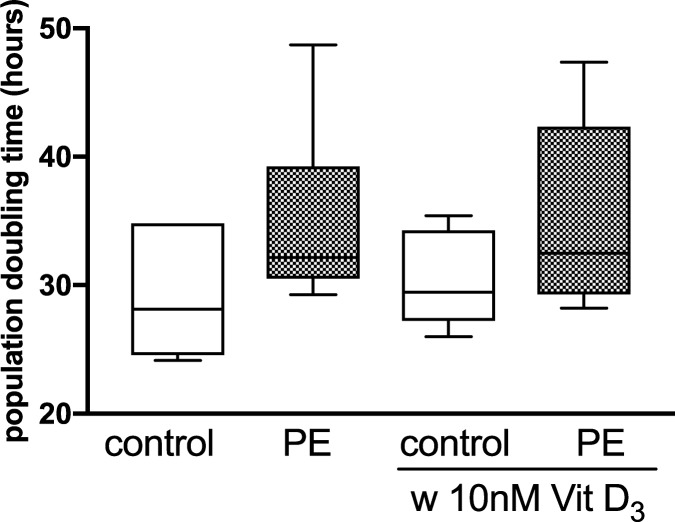
Effect of pregnancy outcome and 1,25(OH)_2_ vitamin D_3_ on HUVEC population doubling time. HUVEC of uncomplicated (control) and preeclamptic (PE) pregnancies were incubated in the presence and absence of 1,25(OH)_2_ vitamin D_3_ (10 nM). Cell numbers were counted and population doubling time calculated after 72 h. The ends of the whiskers represent the maximum and minimum measured values. Distribution was examined using Kolmogorov- Smirnov test. Continuous data were compared with Mann-Whitney test.

The population doubling time (proliferation) of HUVEC isolated from preeclamptic pregnancies was only marginally imparied (34.8 h) compared to uncomplicated pregnancy (29.7 h) during 72 h of culture (P = 0.16) (Fig 4). Concentrations of 10 nM 1,25(OH)_2_ vitamin D_3_ did not change the doubling time of uncomplicated or preeclamptic pregnancies.

### RT-PCR for quantification of the VEGF gene expression

**Fig 5:**
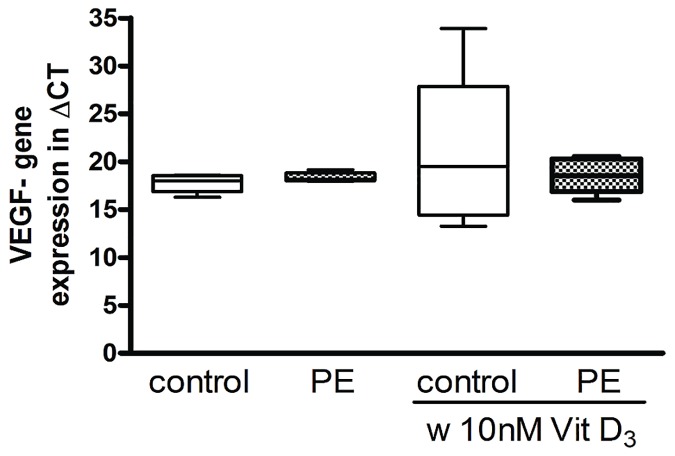
VEGF gene expression of HUVEC from uncomplicated (control) and preeclamptic (PE) pregnancies in the presence and absence of 1,25(OH)_2_ vitamin D_3_ (10 nM) in ug/ml. For each treatment medium triplets were created and three RT-PCR runs were performed for each patient. The ends of the whiskers represent the maximum and minimum measured values. Distribution was examined using Kolmogorov- Smirnov test. Continuous data were compared with Mann-Whitney test.

There was no significant difference in the VEGF gene expression of HUVEC from healthy pregnancies compared to HUVEC from peeclamptic pregnancies (P = 0.39) (Fig 5). The treatment with 10nM 1,25(OH)_2_ vitamin D_3_ did not change the VEGF gene expression neither in the control group (P = 0.37) nor in the preeclamptic group (P = 0.75).

## Discussion

Adverse changes of the cardiovascular system in offspring of women with preeclampsia have been described already in childhood and in young adulthood. However, at the time of birth when most babies appear to be healthy and classical risk factors for cardiovascular disease (e.g. high BMI, smoking, hypertension) are not present, merely the maternal history and the pregnancy outcome are used to predict an infant’s risk for chronic disease. Therefore, we thought to use a fetal endothelial cell model (HUVEC), to examine if the effect of preeclampsia on endothelial function can be detected already this early in life and if a therapeutic intervention using 1,25(OH)_2_ vitamin D_3_ can improve cell characteristics.

In line with our hypothesis we showed a negative impact of preeclampsia on functional characteristics, e.g. tubule formation and migration, of endothelial cells derived from the umbilical vein. A similar effect on migration was observed after treatment of HUVEC with CM derived from placental explant cultures at 2% O_2_ compared to NCM. To our knowledge we are the first to who show a neutral effect of 1,25(OH)_2_ vitamin D_3_, which is in contrast to previous studies, in which the impaired migration, proliferation and tubule formation of fetal, cord blood-derived endothelial progenitor cells from preeclamptic pregnancies was improved [9, 10, 21].

Placental vasculogenesis and angiogenesis are important for a healthy pregnancy. In preeclampsia various angiogenic abnormalities in the feto-placental unit and alterations of circulating angiogenic factors have been described [25, 26]. Our finding here of a reduced average tubule length under standard culturing conditions (21% O_2_) in an *in vitro* angiogenesis model in preeclampsia is consistent with a functional study that described inherent differences of the angiogenic capacity in HUVEC from women with the disease [27]. The migration of endothelial cells marks an important contribution to angiogenesis. Therefore, impaired migration and tubule formation as observed in HUVEC of preeclamptic pregnancies in this study may be germane to angiogenic abnormalities observed in preeclampsia. Although the generation times of HUVEC from preeclamptic pregnancies were 17% higher they did not statistically differ from the generation times of HUVEC from healthy patients.

Incompletely remodeled spiral arteries and the associated dysfunctional blood flow are central features in the pathophysiology of preeclampsia [28, 29]. Fluctuating oxygen concentrations lead to oxidative stress and the release of inflammatory cytokines, antiangiogenic factors and reactive oxygen species, that target endothelial cells causing generalized maternal endothelial dysfunction [30–35]. To simulate these non-physiologic conditions *in vitro* and to assess the effect of aberrant oxygen conditions on endothelial cell migration we used 25% v/v of placental explant CM obtained from incubations at 2% O_2_ (to mimic placental hypoxia in the second half of pregnancy), 8% O_2_ (to approximate placental normoxia in the second half of pregnancy) and 21% O_2_ (standard culture conditions). To the best of our knowledge this is the first study to show that hypoxic placental CM induced a reduction in HUVEC migration. Previously, Moyes et al. described a significant reduction of tubule formation of HUVEC *in vitro* incubated at 0.5% O_2_. *[27].* Our findings are in line with a report of decrease in endothelial cell ATP levels, mitochondrial dehydrogenase activity, and mitochondrial membrane potential and increased endothelial cell death with medium conditioned by hypoxic explants [34]. However, direct exposure to hypoxia of HUVEC [36], human breast cancer cells [37] or murine stem cells [38] stimulated cell migration. These results are in contrast to our findings but could be explained by different effects of direct hypoxia that stimulates angiogenesis compared to hypoxic CM that we deployed. Hypoxic CM contains potentially pathogenic factors, e.g. sFlt-1 and inflammatory cytokines [39] implicated in the endothelial cell dysfunction of preeclampsia. Moreover, chronic hypoxia as employed in some of the studies might not exactly model the effects of fluctuations (hypoxia-reoxygenation) thought to frequently occur with preeclampsia [40].

Vitamin D deficiency is associated with adverse cardiovascular outcomes and an increased risk for the development of preeclampsia (14,15,17,18,19). Compared with uncomplicated pregnancies, there is a reduced maternal, placental and fetal vitamin D pool [41]. Although in this *in vitro* study the overall patient numbers were low (N = 12 and 13) we confirmed significantly lower maternal serum concentrations of 25(OH) vitamin D in preeclamptic participants. Interestingly, although not statistically different fetal cord blood concentrations in preeclampsia were 63% lower compared to healthy pregnancies. This in accordance with previous findings [10]. Four out of 12 preeclamptic cases were early-onset preeclampsia < 34 weeks gestation. While early cases are often clinically more severe and one could expect a different experimental response, we performed a subanalysis of our data. Here, we didn’t see a clear pattern of a stronger impairment of HUVEC functional characteristics in early-onset cases compared to late-onset preeclampsia.

In contrast to our hypothesis we did not confirm a significant effect of 1,25 (OH)_2_ vitamin D_3_ on endothelial cell function. Neither HUVEC of healthy nor HUVEC from preeclamptic pregnancies changed their angiogenic, migratory or proliferative behaviour when incubated with 10 nM 1,25 (OH)_2_ vitamin D_3_. Previous studies on the effect of 1,25 (OH)_2_ vitamin D_3_ yielded controversial results. While a negative effect of 1,25 (OH)_2_ vitamin D_3_ on endothelial cells isolated from the bovine aorta and a decrease of migration of endothelial cells from the pulmonary artery [42] have been found [43], an increased migration of vascular smooth muscle cells (VSMC) [44] and no effect on the migration of retinal endothelial cells [45] have been described. Our previous work showed a significant inhibitory effect of preeclampsia, preeclamptic serum or hypoxic CM on fetal ECFC migration, proliferation and tubule formation [9, 10, 21]. In these studies, 1,25 (OH)_2_ vitamin D_3_ rescued fetal ECFC function under the described conditions [9, 10, 21]. ECFC are a subset of endothelial progenitor cells and critical to blood vessel formation and repair [46]. ECFC dysfunction may represent an early risk factor or risk marker for cardiovascular disease [47]. ECFCs reportedly differ from HUVECs or human umbilical artery endothelial cells in the expression of differentiation-related surface markers (such as CD44) or activities such as proliferation or telomerase activities [48]. However, the reason for the distinct proangiogenic response to vitamin D by fetal ECFC is presently unclear. Apparently, ECFC may be more plastic and adaptable and thus capable of responding compared to the more mature and differentiated HUVEC. In addition, angiogenesis could be regulated by different vitamin D receptor cascades, which vary depending on differentiation, localization or species of the cells. Our data in fetal endothelial cell models suggest that the vitamin D effect is mediated via the fetal progenitor endothelial cells and not via mature fetal endothelial cells.

The effect of preeclampsia on VEGF gene expression in HUVEC has not been studied yet. In this study, the VEGF gene expression of HUVEC from preeclampsia was not different from the gene expression of healthy pregnancies. However, an increase of the VEGF receptor expression in preeclamptic placentas [33], an increase of VEGF gene expression in HUVEC under hypoxic conditions [36, 49], and a decrease of free VEGF in fetal hematopoietic EPCs from preeclamptic women [50] was described previously. In contrast to our assumption 1,25 (OH)_2_ vitamin D_3_ did not affect VEGF gene expression in HUVEC from either healthy nor preeclamptic pregnancies. Cardus et al. showed a stimulating effect of 1,25 (OH)_2_ vitamin D_3_ on VEGF gene expression in VSMC and others confirmed this finding in embryo fibroblasts [51], osteoblasts [52] and fetal ECFC [21].

At the time of birth the umbilical cord and cord blood provide easy accessible tissue and cells. ECFC and HUVEC of the developing fetus are exposed to an adverse metabolic milieu during preeclamptic pregnancies. They might serve as a source for the development of predictive models for later life disease risk. However, at this point it is unclear, if the observed impairments of cell function and metabolism persist throughout child- and adulthood or if they are a temporary effect of the acute disease. Therefore, long-term follow-up studies need to assess the feasibility of using these vascular cells for risk prediction and if an association of functional observations with other clinical measures of endothelial dysfunction exists. Since cardiovascular events remain the main cause of death in the developed world the identification of offspring at risk for cardiovascular alterations as early in life as possible is urgently needed and will allow for closer observation and risk-reducing interventions already in early childhood.
